# The epigenetic regulator Histone Deacetylase 1 promotes transcription of a core neurogenic programme in zebrafish embryos

**DOI:** 10.1186/1471-2164-12-24

**Published:** 2011-01-12

**Authors:** Michael RM Harrison, Aristophanes S Georgiou, Herman P Spaink, Vincent T Cunliffe

**Affiliations:** 1 MRC Centre for Developmental and Biomedical Genetics, Department of Biomedical Science, University of Sheffield, Western Bank, Sheffield S10 2TN, United Kingdom; 2 Institute of Biology, University of Leiden, Wassenaarseweg 64, Leiden, 2333 AL, The Netherlands; 3 Current Address: Saban Research Institute Childrens Hospital Los Angeles 4650 Sunset Blvd. MS# 137 Los Angeles, CA 90027, USA; 4 Current Address: Department of Zoology, Tinbergen Building, University of Oxford, South Parks Road, Oxford, OX1 3PS, UK

## Abstract

**Background:**

The epigenetic regulator Histone Deacetylase 1 (Hdac1) is required for specification and patterning of neurones and myelinating glia during development of the vertebrate central nervous system (CNS). This co-ordinating function for Hdac1 is evolutionarily conserved in zebrafish and mouse, but the mechanism of action of Hdac1 in the developing CNS is not well-understood.

**Results:**

A genome-wide comparative analysis of the transcriptomes of Hdac1-deficient and wild-type zebrafish embryos was performed, which identified an extensive programme of gene expression that is regulated by Hdac1 in the developing embryo. Using time-resolved expression profiling of embryos, we then identified a small subset of 54 genes within the Hdac1-regulated transcriptome that specifically exhibit robust and sustained Hdac1-dependent expression from early neurogenesis onwards. 18 of these 54 stringently Hdac1-regulated genes encode DNA-binding transcription factors that are implicated in promoting neuronal specification and CNS patterning, including the proneural bHLH proteins Ascl1a and Ascl1b, as well as Neurod4 and Neurod. Relatively few genes are strongly repressed by Hdac1 but expression of the Notch target gene *her6 *is attenuated by Hdac1 in specific sub-regions of the developing CNS, from early stages of neurogenesis onwards. Selected members of the stringently Hdac1-regulated group of genes were tested for Hdac1 binding to their promoter-proximal *cis*-regulatory elements. Surprisingly, we found that Hdac1 is specifically and stably associated with DNA sequences within the promoter region of *ascl1b *during neurogenesis, and that this Hdac1-*ascl1b *interaction is abolished in *hdac1 *mutant embryos.

**Conclusions:**

We conclude that Hdac1 regulates histone acetylation and methylation in the developing zebrafish embryo and promotes the sustained, co-ordinate transcription of a small set of transcription factor genes that control expansion and diversification of cell fates within the developing CNS. Our *in vivo *chromatin immunoprecipitation results also suggest a specific function for Hdac1 in directly regulating transcription of a key member of this group of genes, *ascl1b*, from the beginning of neurogenesis onwards. Taken together, our observations indicate a novel role for Hdac1 as a positive regulator of gene transcription during development of the vertebrate CNS, in addition to its more well-established function in transcriptional repression.

## Background

Histone modifying enzymes are key catalytic components of the transcriptional control systems that programme multicellular development. Many different histone modifying enzymes contribute to the dynamic regulation of chromatin structure and function, with concomitant impacts on gene transcription. For example, the balance of Histone acetyltransferase (HAT) and Histone deacetylase (HDAC) activities that are associated with any given gene determines the distribution of histone acetylation marks in the chromatin domain encompassing that gene. Histone acetylation is a hallmark of transcriptionally active chromatin, whereas transcriptionally silent chromatin lacks this modification [1]. Mechanistic analysis of protein complexes that establish and maintain transcriptional repression has revealed the presence of HDACs in these complexes [2,3]. Whilst there is much evidence in support of functions for HDACs in transcription silencing, the roles of HDACs in facilitating transcription have been less well appreciated. Nevertheless, some genome-wide studies in yeast have demonstrated that HDACs are associated with transcriptionally active genes and that they promote gene transcription [4-6]. More recently, mammalian HDACs have been shown to be specifically enriched in chromatin encompassing the transcriptional start sites of transcriptionally active genes, as well as at transcriptionally silent genes that are poised for activation [7]. Moreover, HDAC-containing protein complexes such as REST/CoREST have been demonstrated to poise transcriptionally silent genes in a specific configuration in neural progenitors, which facilitates their robust transcriptional activation when these cells are induced to differentiate into neurons [8].

In zebrafish, the Class I HDAC, Hdac1, is required for specification of neurones and glia during embryonic development [9-12]. In addition, prominent roles are known for this gene in the development of the gastrointestinal system and neural crest derivatives [13-15]. In the mouse, there are two murine orthologues of zebrafish *hdac1*, *Hdac1 *and *Hdac2*, which together promote the transformation of embryonic neural progenitors into neurones and glia [16,17]. In both zebrafish and mouse, Hdac1 regulates neural progenitor differentiation by facilitating the integration of Hedgehog, Notch and Wnt signalling pathway activities into the mechanisms governing neuronal and glial specification. However, precisely how Hdac1 accomplishes this role is still not well understood.

The establishment of proneural gene expression patterns in early embryonic ectoderm delineates zones of active neurogenesis within the neural plate [reviewed in [18]]. Proneural gene expression is repressed by the Notch pathway as a result of negative feedback from Delta-expressing neuronal precursors, which activates Notch in proliferating neural progenitors, thus limiting the rate at which these cells are transformed into neuronal precursors [reviewed in [19]]. Whilst this negative feedback mechanism has been well-characterised, the mechanisms that positively regulate expansion and diversification of differentiated cell types within the nervous system are less well understood. Our previous studies demonstrated that Hdac1 promotes expression of proneural genes, represses Notch target gene expression and enables neural fate-determining responses to Hedgehog pathway activity [9,12]. In view of the well-documented roles of HDACs as components of transcriptional repressor complexes, these observations suggested a role for Hdac1 as a potential direct repressor of *her6 *transcription, and thus as an indirect activator of proneural genes. Here, we describe the results of our recent work to elucidate further the role of Hdac1 in neurogenesis, using global gene expression profiling tools to define the Hdac1-regulated transcriptome in an unbiased way and to identify direct targets of Hdac1 using chromatin immunoprecipitation.

## Results

### Identification of Hdac1-regulated genes by transcriptome analysis of *hdac1 *mutant zebrafish embryos

**Figure 1:**
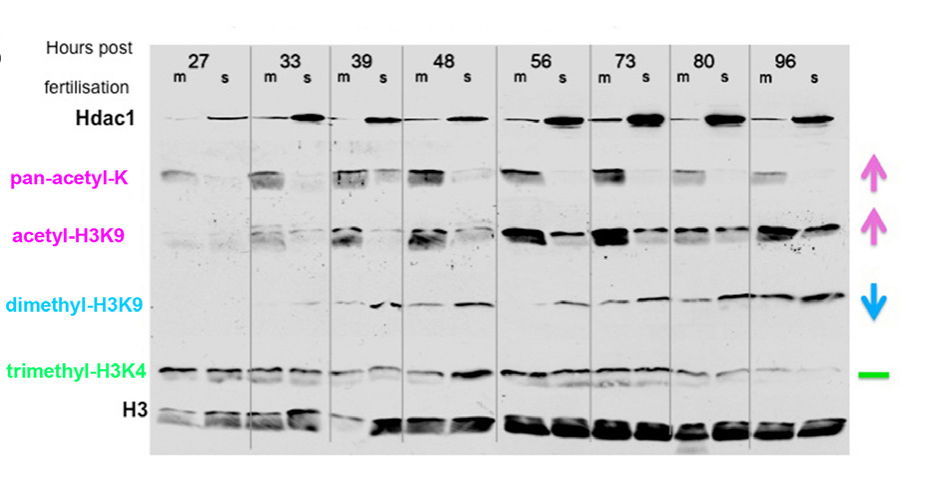
**Hdac1 inhibits Histone acetylation and promotes H3K9 methylation levels in the zebrafish embryo**. Levels of Hdac1 protein and covalently modified histones in *hdac1 *mutant (m) and wild-type (s) embryos aged 27, 33, 39, 48, 56, 73, 80 and 96 hpf, visualised by Western blot analysis using anti-Hdac1 and histone modification-specific antibodies. Hdac1 protein visualised with an anti-Hdac1 antibody is shown on the top row. Antibodies specific for the following histone modifications were: Pan-acetyl lysine; acetyl-H3K9; dimethyl-H3K9, trimethyl-H3K4. An anti-H3 antibody was used as a loading control (bottom row).

Previous studies of the *hdac1 *mutant embryonic CNS revealed extensive defects of neuronal specification and patterning, along with an absence of differentiated oligodendrocytes [9-12]. When the levels of Hdac1 protein, histone acetylation and histone methylation were compared in *hdac1 *mutant and wild-type sibling embryos between 27 hpf and 96 hpf, *hdac1 *mutants exhibited consistently reduced levels of Hdac1 protein, together with a large, persistent increase in global histone acetylation and a stable reduction in H3K9 methylation, in comparison to wild-type siblings (Figure 1). By contrast, levels of H3K4 methylation were mostly unaffected by loss of *hdac1 *function. The major differences in the abundance of acetylated histones and methylated H3K9, that were detected between *hdac1 *mutant and wild-type sibling embryos, demonstrated that Hdac1 plays a key role in the epigenetic regulation of chromatin structure, and implied that there would be major impacts of Hdac1 function on transcriptome composition. Therefore, to develop a systems-level understanding of Hdac1 function during embryonic development, we used a global gene expression profiling platform to identify the Hdac1-regulated transcriptome in developing zebrafish embryos. By defining temporal changes in the composition of the Hdac1-regulated transcriptome from the beginning of neurogenesis onwards, we sought to identify genes whose normal transcript levels exhibited a continuous dependence on Hdac1 function throughout neurogenesis, and which could thus be important direct effectors of Hdac1 function in the developing CNS.

**Figure 2:**
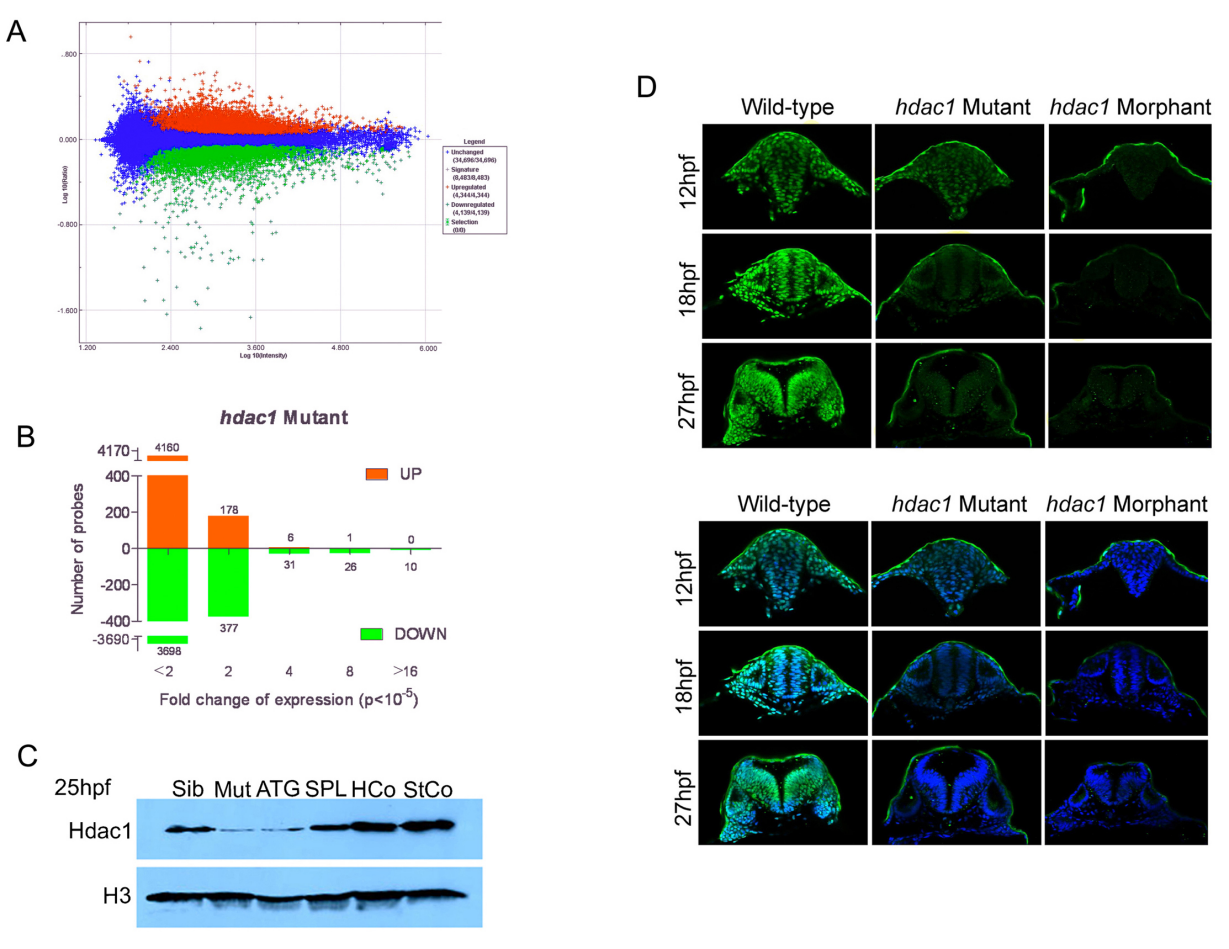
**Phenotypic analysis of Hdac1-deficient embryos**. (A) Scatter plot of probe intensities from *hdac1 *mutant versus sibling comparisons. Up-regulated and down-regulated probes in *hdac1 *mutants are coloured red or green, respectively. The averaged ratio values are plotted on the y-axis and the averaged expression values are plotted on the x-axis, both of which are expressed in a log_10 _scale. The threshold for statistically significant differential expression was set at p < 10^-5^. (B) Analysis of the fold-change distribution of Hdac1-regulated probes identified in (A). (C) Microinjection of Hdac1 morpholinos reduces Hdac1 protein abundance in zebrafish embryos. Western blotting for Hdac1 protein in *hdac1 *mutant (Mut), wild-type (Sib), Hdac1ATG1 morphant (ATG), Hdac1SPL1 morphant (SPL), Hdac1Control morphant (HCo) and Standard Control morphant (StCo) embryos. (D) Reduced immunostaining for Hdac1 in *hdac1 *mutant and Hdac1ATG1 morphant embryos. Transverse confocal sections through the hindbrain of 12 hpf, 18 hpf and 27 hpf wild-type, *hdac1 *mutant and Hdac1ATG1 morphant embryos immunostained for Hdac1 protein (green in upper and lower panels) and counterstained with DAPI (blue, in lower panel only).

As a starting point, 27 hpf *hdac1 *mutant and wild-type sibling embryos were sorted into morphologically distinct groups. RNA was extracted from sorted embryos, labelled and hybridised to Agilent arrays comprising 43427 oligonucleotide probes, which corresponded to 18636 unique Unigene clusters and approximately 2000 additional Ensembl-annotated genes [20]. Using a p-value threshold of <10^-5 ^to define statistically significant expression differences between *hdac1 *mutant and wild-type sibling samples, we observed that 4345 probes exhibited increased expression in the 27 hpf *hdac1 *mutant samples, whereas 4142 probes exhibited decreased expression in these samples (Figure 2A, B). 629 of these Hdac1-regulated probes exhibited a 2-fold or greater increase or decrease in transcript abundance as a result of the *hdac1 *mutation.

### Hdac1-targeted morpholinos closely phenocopy the transcriptional defects exhibited by *hdac1 *mutant embryos

Comparison of *hdac1 *mutant and wild-type transcriptomes at 27 hpf identified large numbers of Hdac1-regulated genes, many of which were likely to be indirect, downstream effectors of the primary target genes for Hdac1. To identify candidate Hdac1 direct target genes, we sought to identify genes whose expression was regulated by Hdac1 during early developmental stages that preceded the emergence of a morphologically recognisable *hdac1 *mutant phenotype. Since morpholinos can be used to create defined batches of embryos that are all deficient in a specific gene function, we compared the phenotypes of embryos microinjected with a translation-blocking morpholino, Hdac1ATG1 [9] or a splice-blocking morpholino Hdac1SPL1, with the phenotype of *hdac1 *mutant embryos. Microinjection of either Hdac1ATG1 or Hdac1SPL1 morpholinos into embryos caused morphological abnormalities that closely resembled the *hdac1 *mutant phenotype (data not shown), and which were first detectable at ~24 hpf. Moreover, both the Hdac1ATG1 and Hdac1SPL1 morpholinos significantly reduced Hdac1 protein levels in the embryo, although the Hdac1ATG1 morpholino induced a larger reduction in Hdac1 protein levels than did the Hdac1SPL1 morpholino (Figure 2C). By contrast, neither an Hdac1 Mismatch Control morpholino (Hdac1 Control), nor a Standard Control morpholino, exhibited any appreciable effect on embryo morphology, or on levels of Hdac1 protein (Figure 2C).

In order to begin to elucidate the effects of Hdac1ATG1 and Hdac1SPL1morpholinos on embryonic transcription, the transcriptomes of these morphant embryos were analysed at 27 hpf. Both the Hdac1ATG1 and Hdac1SPL1 morpholinos elicited major effects on the embryonic transcriptome (Additional file 1). A set of 6117 probes was identified, from within the group of probes whose expression is affected in the *hdac1*mutant, that also exhibited statistically significant (p < 10^-5^) Hdac1-regulated gene expression in 27 hpf morphant samples. This set of 6117 probes was further analysed with the Explore Gene Ontology (eGOn) software, to identify Gene Ontology (GO) categories that were significantly enriched in the Hdac1-regulated probe set (Additional file 2). The Biological Process category was found to be considerably over-represented in the annotations of Hdac1-regulated genes, within which the sub-categories of GO terms related to Transcriptional Regulation (highlighted in purple, Additional file 2) or Developmental Processes occurred particularly frequently. Other prominent GO terms that are enriched in the Hdac1-regulated probe set include nervous system development, neuronal differentiation and photoreceptor differentiation (Additional file 2). Thus, both *hdac1 *mutant and morphant transcriptomes exhibit similar alterations to the expression of genes that encode transcription factors and developmental regulators, with an emphasis on development of the nervous system. These results implied that Hdac1 performs a central role in regulating expression of these classes of genes and further validated the morpholinos as tools for perturbing Hdac1 function during nervous system development.

In order to determine which of the two Hdac1 morpholinos most closely phenocopied the *hdac1 *mutation, hierarchical cluster analysis [21] was used to order the transcriptome datasets obtained from 27 hpf *hdac1 *mutant, Hdac1ATG1 and Hdac1SPL1 morphant embryos, according to the similarities in the patterns of gene expression changes observed in each of the three different manipulations (Additional file 1). This analysis showed that overall, the set of Hdac1-regulated genes that was identified using the Hdac1ATG1 morpholino, more closely resembled the group of genes identified by analysis of 27 hpf *hdac1 *mutant embryos than did those identified using the Hdac1SPL1 morpholino. The Hdac1ATG1 morpholino was therefore chosen to investigate Hdac1 function during early stages of CNS development before a morphologically distinct phenotype can be recognised. As a first step in this analysis, we determined whether this morpholino could reduce Hdac1 protein levels from early stages of CNS development onwards. Hdac1ATG1 morphant embryos and individually genotyped wild-type and *hdac1 *mutant embryos, aged 12 hpf, 18 hpf and 27 hpf (corresponding to early, mid- and late neurogenesis stages, respectively) were immunostained with an anti-Hdac1 antibody and analysed by confocal imaging (Figure 2D). Whilst Hdac1 protein was abundant in all cell nuclei of wild-type embryos at each of the stages analysed, both *hdac1 *mutant and Hdac1ATG1 morphant embryos exhibited a substantially lower level of Hdac1 protein, which was more pronounced at later developmental stages (Figure 2D). The results of the transcriptomic analysis, together with the experiments to compare the distribution of Hdac1 protein in *hdac1 *mutant and morphant embryos, revealed a close correspondence between the 27 hpf *hdac1 *mutant and morphant phenotypes and validated the Hdac1ATG1 morpholino as a precision tool with which to investigate Hdac1 function in the embryo between 12 hpf and 27 hpf.

### Hdac1 promotes expression of a core set of neurogenic regulatory genes throughout the developing CNS

**Figure 3:**
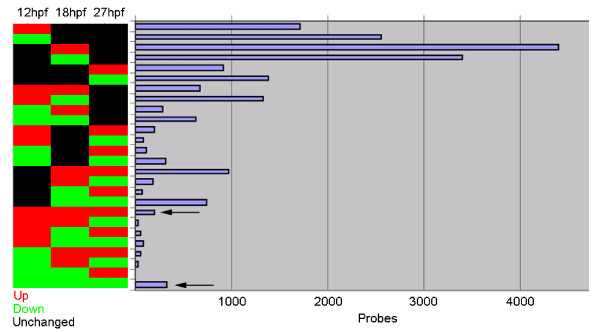
**Time-resolved transcriptome analysis identifies an Hdac1-regulated gene expression programme that is sustained throughout neurogenesis**. Cluster3.0 analysis of the transcriptomes of Hdac1ATG1 and Standard Control (StCo) morphant embryos at 12, 18 and 27 hpf. Up-regulated probes are indicated in red; down-regulated probes are indicated in green; unchanged probes are indicated in black. The threshold for statistically significant differential expression was set at p < 10^-5 ^and an additional fold-change minimum of 1.5 was also imposed. All probes on the array are grouped based on their expression changes across the three time points. Large numbers of gene changes were identified as being misregulated at individual time points, however 803 probes were found to be misregulated at all three time points. Only 199 probes were consistently up-regulated (red, arrow) and 335 probes were found to be down-regulated (green, arrow). Probes that exhibited sustained fold-changes in expression >2-fold across all three time points are listed and annotated in Additional file 3, of which those that also exhibited altered expression in *hdac1 *mutant embryos are listed in Figure 4.

To identify genes whose expression was regulated by Hdac1 during early stages of neurogenesis, pools of embryos were prepared that were injected with either the Hdac1ATG1 morpholino or the Standard Control morpholino, and samples were collected at 12 hpf and 18 hpf for RNA extraction and analysis. At 12 hpf, neurogenesis has just begun, by 18 hpf it is well underway, and by 27 hpf this process is widespread throughout the nervous system. Transcriptomes were analysed and the Hdac1-regulated gene datasets were compared to those obtained from analysis of 27 hpf Hdac1ATG1 morphants (Figure 3). The results of this cluster analysis revealed that considerably more differential gene expression was detectable between the transcriptomes of Hdac1ATG1 and Standard Control morphant embryos at 12 hpf and 18 hpf, than was detectable at 27 hpf, even though there are no appreciable morphological abnormalities in Hdac1ATG1 morphant embryos before 22 hpf.

**Figure 4:**
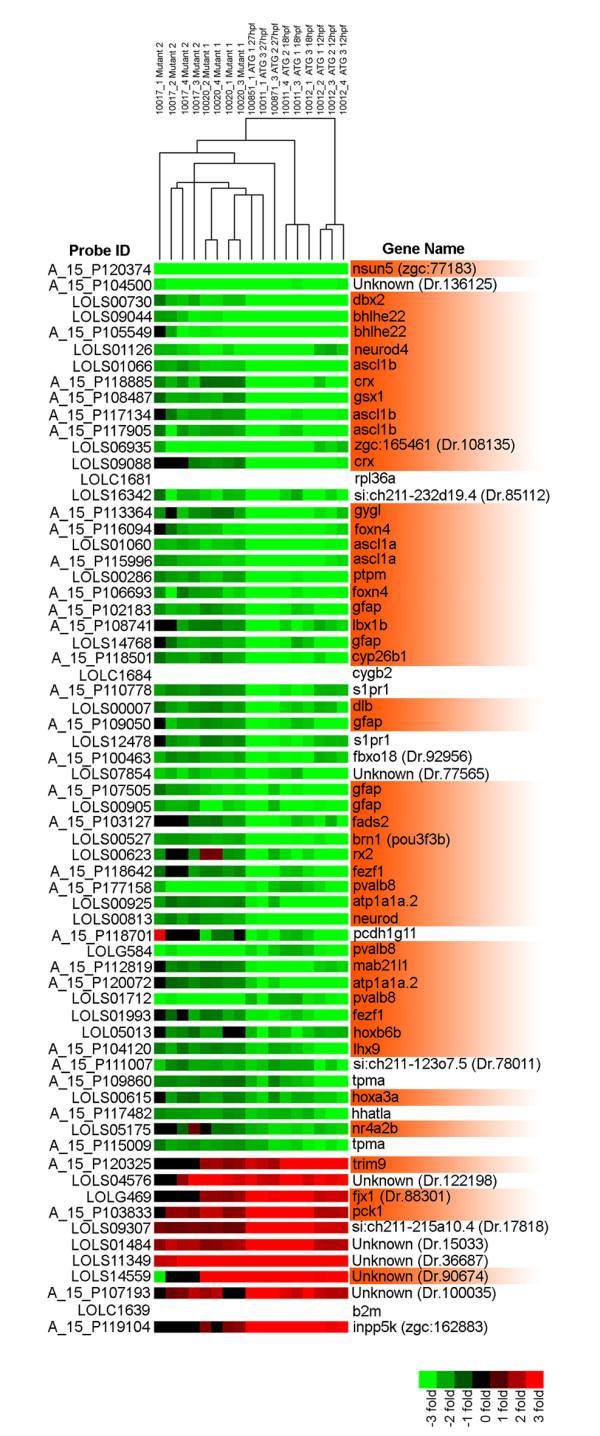
**Cluster analysis of *hdac1 *mutant and morphant transcriptomes identifies a core set of Hdac1-regulated genes**. The Cluster Tree displays the relationships between transcriptomes of 27 hpf *hdac1 *mutant embryos and 12 hpf, 18 hpf and 27 hpf Hdac1ATG1 morphant embryos. The threshold for statistically significant differential expression was set at p <10^-5 ^for all probes and an additional >2-fold-change criterion was then applied to identify a subset of 84 probes whose expression was changed in Hdac1ATG1 morphants at all three times points (Suppl. Table S2). To eliminate probes from this group that did not exhibit altered expression in the 27 hpf *hdac1 *mutant samples, an exclusion criterion was then applied to eliminate probes that did not exhibit a >1.2-fold change in transcript abundance in 27 hpf *hdac1 *mutants in comparison to controls. Thus, 67 probes were identified that exhibited a >2-fold change in transcript abundance in Hdac1ATG1 morphants at 12 hpf, 18 hpf and 27 hpf and a >1.2 fold change in transcript abundance in *hdac1 *mutants, in comparison to controls. All of these expression changes met the threshold for statistical significance of p < 10^-5^. The expression changes for each of the listed genes are given for each of the individual microarrays analysed. Up-regulated probes are indicated in red; down-regulated probes are indicated in green. The samples include 8 *hdac1 *mutant/wild-type sibling replicates, plus biological triplicates for each of the 12 hpf, 18 hpf and 27 hpf Hdac1ATG1/Standard Control morphant comparisons (see Methods section for further details). Genes that are expressed in the CNS or have a CNS-oriented function are indicated in orange.

The cluster analysis identified several discrete gene classes with temporally distinct, differential expression profiles in Hdac1ATG1 and control morphant embryos, including a small subset of genes that exhibited consistent, statistically significant transcriptional sensitivity to loss of Hdac1 at all three stages analysed (arrows in Figure 3). Thus, in Hdac1ATG1 morphant embryos, 199 probes were up-regulated at 12 hpf, 18 hpf and 27 hpf, whereas 335 probes were down-regulated at all three time points, in comparison to their expression levels in Standard Control morphants. In order to identify the genes exhibiting the greatest degree of sustained, differential gene expression at all three time points, probes exhibiting a >2-fold change in expression level at all three time points were selected. 84 of these probes exhibited a >2-fold change in expression in Hdac1ATG1 morphants at all three time points analysed (Additional file 3). However, in view of the fact that morpholinos can sometimes elicit off-target effects that are not recapitulated by a corresponding mutant, we applied an additional exclusion criterion to eliminate probes that exhibited less than a 1.2-fold change in expression (of the same polarity) in 27 hpf *hdac1 *mutants, as compared to wild-type embryos (Additional file 3). Applying this criterion removed 17 of the 84 probes that exhibited differential expression in Hdac1ATG1 and Standard Control morphants, thus reducing the number of probes in this group to 67. Of these Hdac1-regulated probes, 56 exhibited decreased transcript abundance in *hdac1 *mutant and Hdac1ATG1 morphant embryos at each of the time points analysed, whereas 11 of the 67 probes exhibited consistent increases in transcript abundance (Figure 4). The 56 down-regulated probes correspond to 43 distinct down-regulated genes, of which 28 are specifically expressed in the CNS or have a known CNS-oriented function. Of these 28 genes, 18 encode transcription factors with known or likely roles in programming of CNS development: *dbx2, bhlhe22, neurod4, ascl1a, ascl1b, crx, gsx1, foxn4, lbx1b, pou23, rx2, fezf1, neurod, mab21l1, hoxb6b, lhx9, hoxa3a, *and *nr4a2*. In addition, the *dlb *gene, encoding the nervous system-specific Notch ligand, DeltaB, is another robustly Hdac1-dependent gene. Other genes implicated in CNS development that were down-regulated in Hdac1-deficient embryos include genes encoding the Retinoic Acid-metabolising enzyme Cyp26b1, GFAP, Fads2 and Atp1a1a.2. The two genes exhibiting the greatest fold reduction in transcript abundance in Hdac1ATG1 morphants across the three time points analysed are *nsun5*, which encodes a protein of unknown, potentially nuclearly-localised function, with a putative methyltransferase-encoding NOL1/NOP2/Sun domain, and a novel gene with no significant sequence similarity to known genes (Unigene ID Dr.136125). Of the 11 up-regulated probes, 4 are specifically expressed in the CNS or have a known CNS-oriented function, such as *trim9 *and *fjx1*, but none encode known transcription factors. Taken together, these results demonstrate that Hdac1 promotes the co-ordinated expression of a core set of neurogenic transcriptional regulators, which we define here as the Hdac1 neurogenic programme, from early stages of neurogenesis onwards. These results are fully consistent with the Gene Ontology analysis of the *hdac1 *mutant (Additional file 2), which identified genes involved in transcriptional control and developmental mechanisms, including nervous system development, as the principal targets of Hdac1-mediated transcriptional regulation.

**Figure 5:**
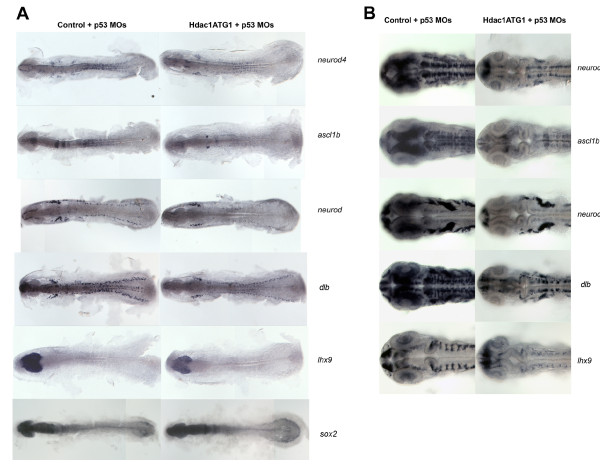
**Expression of the Hdac1 neurogenic programme is stably attenuated in Hdac1ATG1 morphant embryos**. (A) 6-somite stage (12 hpf) embryos and (B) 27 hpf embryos injected with standard Control + p53 morpholinos or Hdac1ATG1 + p53 morpholinos, analysed for expression of *neurod4, ascl1b, neurod, dlb*, *lhx9 *and *sox2*. Expression of *neurod4, ascl1b, neurod, dlb *and *lhx9 *was reduced in Hdac1-deficient embryos. In contrast, the expression pattern of *sox2*, which did not exhibit a statistically significant change in transcript abundance in the microarray analysis, was unaltered in 12 hpf Hdac1ATG1 morphant embryos.

**Figure 6:**
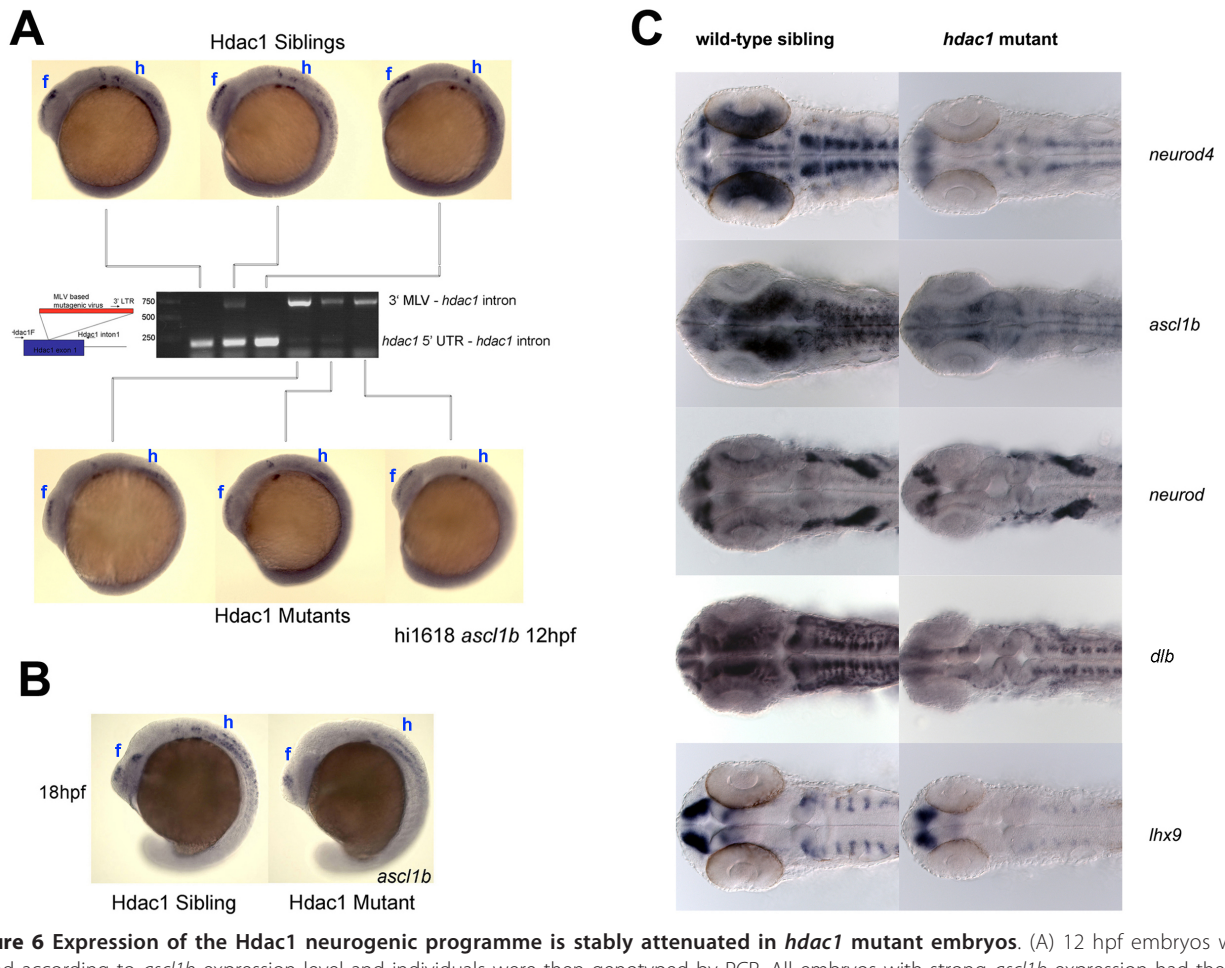
**Expression of the Hdac1 neurogenic programme is stably attenuated in *hdac1 *mutant embryos**. (A) 12 hpf embryos were sorted according to *ascl1b *expression level and individuals were then genotyped by PCR. All embryos with strong *ascl1b *expression had the wild type *hdac1 *allele (lower band) and were therefore wild-type siblings, whereas embryos with a weak *ascl1b *expression pattern carried only the mutant *hdac1 *allele (upper band) and were therefore *hdac1 *mutants. (B) Similar results to those shown in (A) were obtained for 18 hpf embryos. In both (A) and (B), the forebrain (f) and hindbrain (h) regions of Hdac1-regulated *ascl1b *expression are indicated. (C) Expression of the Hdac1 neurogenic programme is attenuated in 27 hpf *hdac1 *mutant embryos. Expression of *neurod4, ascl1b, neurod, dlb *and *lhx9 *in 27 hpf *hdac1*mutant and wild-type sibling embryos. Expression of each of these genes is reduced in the CNS of *hdac1 *mutant embryos.

To document the spatial extent of Hdac1 function in the neural plate during neurogenesis, the expression patterns of a subset of components of the Hdac1 neurogenic programme, *neurod4, ascl1b, neurod, lhx9 *and *dlb*, were characterised by whole-mount *in situ *hybridisation of 6 somite-stage (12 hpf) and 27 hpf Hdac1ATG1 morphant and control morphant embryos (Figure 5). We observed that Hdac1ATG1 morphants exhibited considerably reduced expression of these genes throughout the developing CNS at both 12 hpf and 27 hpf. Coinjection of a p53-targeted morpholino, which eliminates off-target effects caused by morpholino injection, had no observable effects on any of the gene expression patterns analysed (Figure 5). By contrast, the expression pattern of *sox2*, which is expressed widely in the developing nervous system, was unaffected by loss of *hdac1 *function at 12 hpf (Figure 5). Consistent with the observations in Hdac1ATG1 morphant embryos, the expression pattern of *ascl1b *was also appreciably reduced in individually genotyped *hdac1 *mutant embryos at 12 hpf and 18 hpf, in comparison to its expression level in wild-type sibling embryos (Figure 6A,B). When the expression patterns of *neurod4, ascl1b, neurod, lhx9 *and *dlb *were then compared in 27 hpf *hdac1 *mutant embryos, expression of all five genes was dramatically reduced throughout the CNS, as was observed in 27 hpf Hdac1ATG1 morphant embryos (Figure 6C). Taken together, these results suggest that, from early stages of neurogenesis onwards, Hdac1 promotes the co-ordinated transcription of developmental regulatory genes, at the core of which lies a set of transcription factors that play key roles in regulating neuronal specification and patterning throughout the CNS.

**Figure 7:**
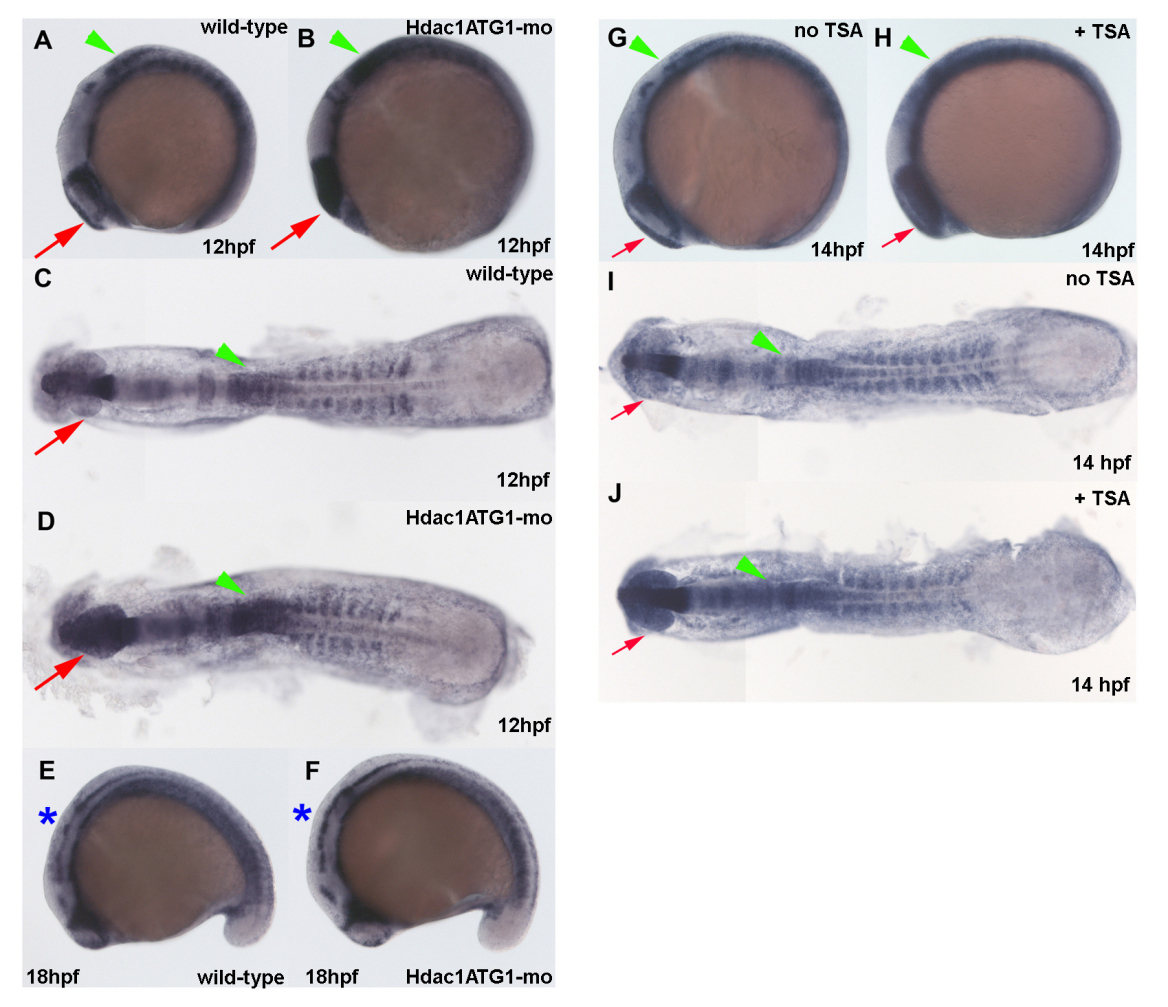
**Increased *her6 *expression in the CNS of Hdac1ATG1 morphants and TSA-treated embryos during early neurogenesis**. (A-F) Wild-type sibling (A, C, E) and Hdac1ATG1 morphant (B, D, F) embryos were analysed for expression of *her6 *at 12 hpf (A, B, C, D) and 18 hpf (E, F). (G-J) Embryos were cultured in the absence (G, I) or presence (H, J) of the HDAC inhibitor TSA (1 μM) from 10-14 hpf, then analysed for expression of *her6*. In dorsal and lateral views, anterior is to the left. Green arrowheads indicate caudal hindbrain, which exhibits increased *her6 *expression in Hdac1ATG1 morphants at 12 hpf and in TSA-treated embryos at 14 hpf; red arrows indicate optic vesicles, which exhibit increased *her6 *expression in Hdac1ATG1 morphants at 12 hpf and in TSA-treated embryos at 14 hpf; blue asterisks indicate hindbrain, which exhibits increased increased *her6 *expression in Hdac1ATG1 morphant embryos at 18 hpf.

Previous studies from our laboratory demonstrated that Hdac1 antagonises Notch signalling and attenuates expression of the Notch target gene *her6 *during CNS development [9]. Moreover, the time-resolved microarray analysis confirmed that statistically significant increases in *her6 *expression could be detected in *hdac1 *mutants and Hdac1ATG1 morphants at 27 hpf, but by contrast, statistically significant changes in *her6 *expression were not detected in 12 hpf and 18 hpf *hdac1 *morphant embryos, which excluded it from the group of robustly Hdac1-regulated genes defined by the microarray analysis (Additional file 3). However, when *her6 *expression was analysed by *in situ *hybridisation, increased expression was observed in the caudal hindbrain and developing optic vesicles of 12 hpf Hdac1ATG1 morphants, and the increased *her6 *expression persisted in the morphant hindbrain at 18 hpf (Figure 7A-F). Thus, although these changes were too small to be reliably detected by the whole-embryo microarray analysis, our *in situ *data show that in discrete regions of the CNS, Hdac1 represses *her6 *transcription from early stages of neurogenesis onwards, which is consistent with previous observations in older embryos [9].

### Pharmacological inhibition of HDAC function during early neurogenesis closely phenocopies the Hdac1ATG1 morphant phenotype

**Figure 8:**
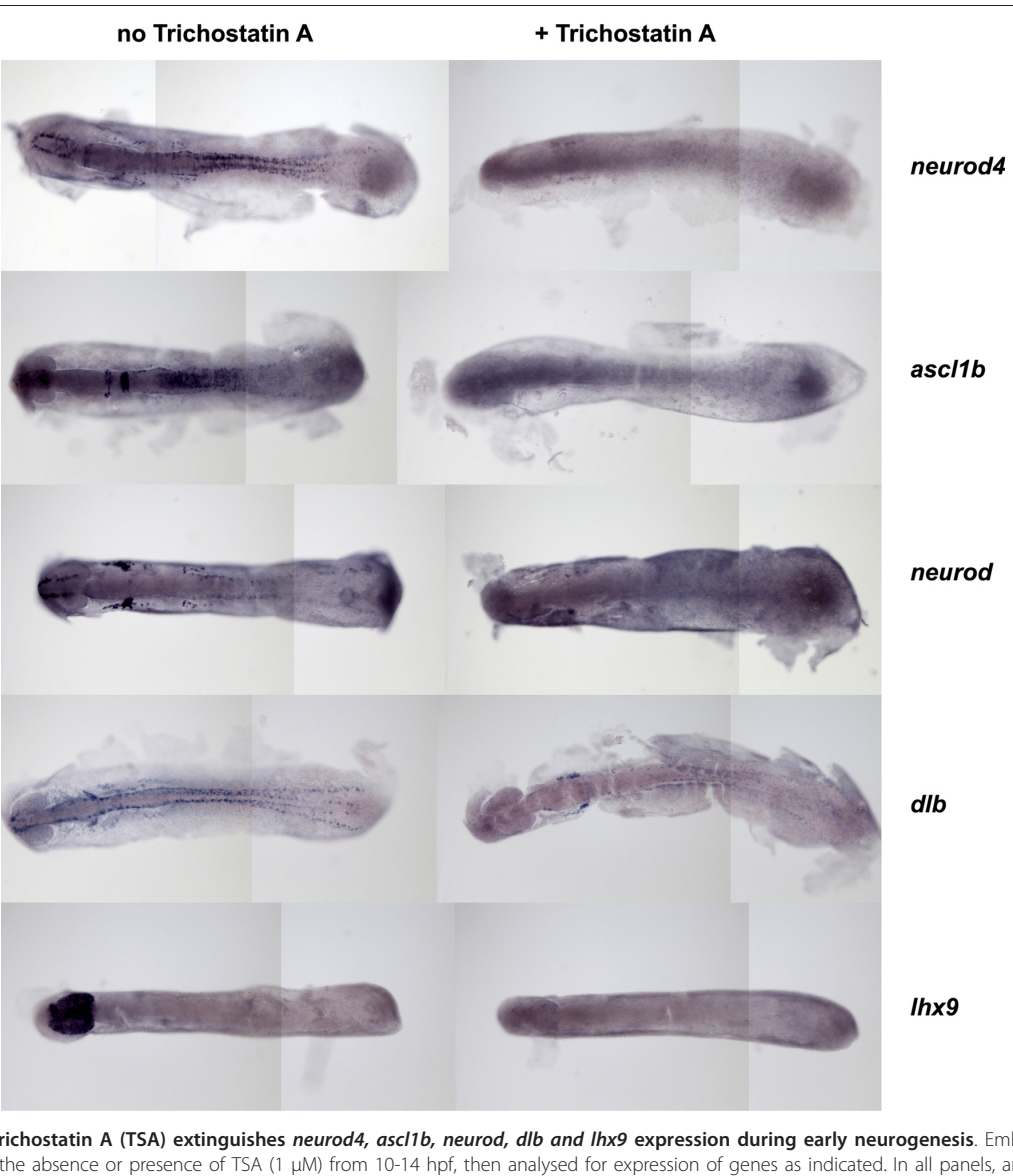
**Trichostatin A (TSA) extinguishes *neurod4, ascl1b, neurod, dlb and lhx9 *expression during early neurogenesis**. Embryos were cultured in the absence or presence of TSA (1 μM) from 10-14 hpf, then analysed for expression of genes as indicated. In all panels, anterior is to the left.

To determine whether HDAC activity is required for correct expression of Hdac1-regulated genes specifically during early neurogenesis, wild-type embryos were incubated with the HDAC inhibitor Trichostatin A (TSA) between 10 hpf and 14 hpf, then fixed and analysed for gene expression by *in situ *hybridisation. These experiments revealed that expression of *neurod4, ascl1b, neurod, dlb *and *lhx9 *in the embryonic CNS was almost completely extinguished after incubation in TSA for 4 hours (Figure 8). Moreover, TSA treatment of embryos from 10-14 hpf caused increased expression of *her6 *in the caudal hindbrain and optic vesicles, as was observed in 12 hpf Hdac1ATG1 morphants (Figure 7G-I). Thus, we conclude that HDACs promote transcription of *neurod4, ascl1b, neurod, dlb *and *lhx9 *throughout the CNS, and attenuate expression of *her6 *in the hindbrain and optic vesicles, during early stages of neurogenesis. In view of the fact that all of these genes are specifically regulated by Hdac1, it seems likely that the effects of TSA during early neurogenesis are mediated via direct inhibition of Hdac1.

### Hdac1 binds directly to the promoter of the proneural gene *ascl1b *in 12 hpf and 27 hpf zebrafish embryos

**Figure 9:**
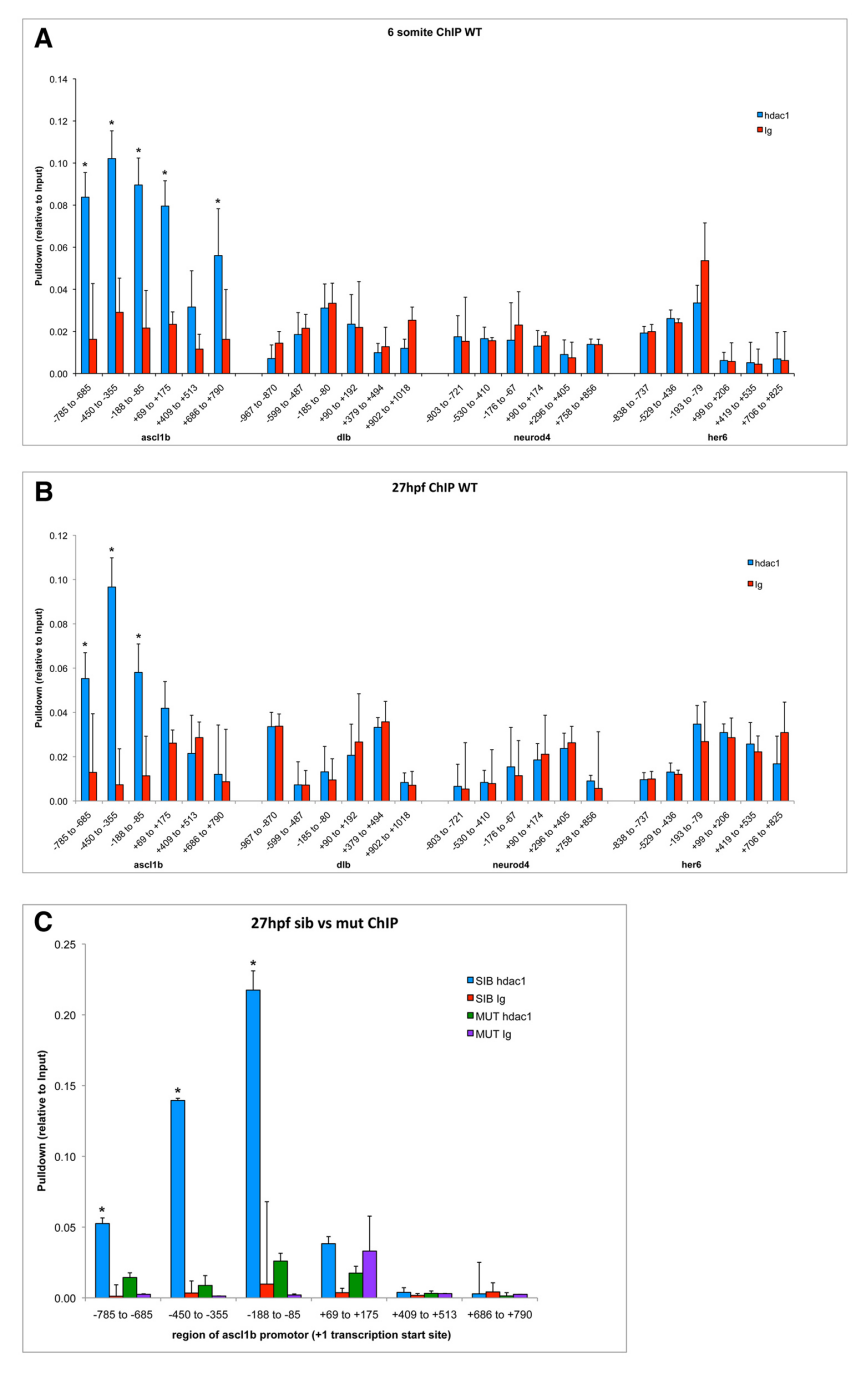
**Chromatin immunoprecipitation analysis of Hdac1 binding to *cis*-regulatory regions of Hdac1-regulated genes**. (A, B) Hdac1 is stably and specifically associated with DNA sequences close to the transcription start site of the *ascl1b *gene in (A) 12 hpf and (B) 27 hpf zebrafish embryos. Chromatin was immunoprecipitated with anti-Hdac1 antibody (blue) and control IgG (red) and DNA content analysed by Q-PCR, which revealed Hdac1 is stably associated with DNA sequences between -785bp and -85bp upstream of *ascl1b *in wild-type embryos. In contrast, Hdac1 was not detectably associated with similarly positioned DNA sequences close to *neurod4, dlb *or *her6*. (C) Immunoprecipitation of *ascl1b *promoter sequences from chromatin with an Hdac1-specific antibody is abolished in 27 hpf *hdac1 *mutant embryos. Comparison of Hdac1 protein binding to chromatin encompassing the *ascl1b *transcription start site in 27 hpf wild-type sibling (SIB) and *hdac1 *mutant (MUT) embryos. Chromatin was immunoprecipitated with anti-Hdac1 antibody (blue, green bars) or control IgG (red, purple bars) and DNA content analysed by Q-PCR. Physical association of Hdac1 with DNA sequences between -785bp and -85bp upstream of *ascl1b *was detected in wild-type sibling embryos, but not in *hdac1 *mutant embryos. Bar graphs show mean values with standard deviations for technical triplicates. Asterisks indicate statistical significance (P < 0.05).

Genome-wide studies of HDAC binding in mammalian T cells have revealed a strong, discrete peak of HDAC1 binding within the chromatin encompassing the transcription start site of many transcriptionally active genes as well as a smaller number of inactive genes, suggesting a direct role for this chromatin modifying enzyme in local control of chromatin structure at both transcribed and silent target gene promoters [7]. The specific enrichment of HDAC1 at the promoters of active genes enables chromatin structure to be reset for subsequent re-acetylation of histones during transcription elongation [7]. Our transcriptomic analysis revealed key roles for Hdac1 in promoting expression of proneural genes and other neural fate-determining factors in the developing CNS, as well as in limiting expression of other genes such as *her6, *within the CNS. Furthermore, we found that pharmacological inhibition of HDAC function caused a rapid shut-down of the neurogenic programme throughout the CNS of 12 hpf embryos, as well as increasing *her6 *expression in restricted regions of the CNS at this stage. We therefore wondered whether Hdac1 might directly bind to the promoter elements of neural fate-determining genes during neurogenesis, in order to directly regulate their transcription. To investigate this possibility, 12 hpf and 27 hpf wild-type embryos were subjected to chromatin immunoprecipitation analysis with the anti-Hdac1 antibody, which was validated to specifically and efficiently immunoprecipitate Hdac1 from embryonic chromatin (Additional file 4). Immunoprecipitated DNA samples were then analysed for the presence of specific sequences within the proximal promoter region, the first exon and the first intron of *ascl1b*, *neurod4*, *deltab *and *her6 *(Figure 9). These four genes were chosen for this analysis because their expression patterns in the developing CNS were consistently Hdac1-regulated between 12 hpf and 27 hpf. Surprisingly, robust and specific binding of Hdac1 was detected throughout the region between -785bp and -85bp upstream of the *ascl1b *transcription start site in both 12 hpf and 27 hpf wild-type embryos (Figure 9A,B). In 12 hpf embryos, Hdac1 binding was also detected in chromatin fragments spanning +69 bp to + 175 bp and +686 bp to +790 bp downstream of the *ascl1b *transcription start site. In striking contrast, no specific binding of Hdac1 to promoter-proximal sequences of *neurod4*, *dlb *or *her6 *was detectable. Comparative analysis of Hdac1 binding to the *ascl1b *promoter region in 27 hpf *hdac1 *mutant and wild-type siblings further revealed that all of the statistically significant Hdac1 binding to the *ascl1b *promoter, in the region between -785bp and -85bp upstream of the *ascl1b *transcription start site, was lost in *hdac1 *mutant embryos (Figure 9C). Our results demonstrate that Hdac1 specifically binds to a region of the *ascl1b *promoter between -785 and -85 bp upstream of the transcription start site and promotes *ascl1b *expression likely in response to neural signals. These results also indicate that no binding of Hdac1 to DNA sequences close to the corresponding transcription start sites of *dlb, neurod4 *or *her6, *could be detected.

## Discussion

### Hdac1 promotes sustained expression of a small subset of developmental regulatory genes from the beginning of neurogenesis onwards

In zebrafish and mouse embryos, Hdac1 promotes the transformation of neural progenitors into differentiated neurons and glia. The experiments reported here further elucidate the mechanism of Hdac1 function in zebrafish CNS development. We initially sought to identify all of the genes whose expression levels were robustly Hdac1-regulated in 27 hpf embryos. We found that approximately 20% of all the probes on the microarrays exhibited statistically significant differences in transcript abundance in 27 hpf *hdac1 *mutant and wild-type sibling embryos, although only 7% of these Hdac1-regulated probes exhibited more than a two-fold change in transcript abundance. However, as this comparative analysis was performed on morphologically distinct 27 hpf *hdac1 *mutants and wild-type siblings, it seemed likely that many of the genes identified in this way as being Hdac1-regulated are indirect downstream effectors of a smaller programme of gene expression that is directly regulated by Hdac1 from earlier stages of neurogenesis. Therefore, to identify transcriptional changes that were likely to be more direct consequences of loss of Hdac1 function, we used the Hdac1ATG1 morpholino to generate defined batches of Hdac1-deficient embryos, whose transcriptomes were then analysed well before any morphological differences appeared in these embryos. Gene Ontology analysis of Hdac1-regulated genes, in both *hdac1 *mutant and Hdac1ATG1 morphant embryos, revealed a consistent and robust enrichment of genes with known functions in both transcriptional control and CNS development. We then discovered within this group a set of 54 genes that exhibited consistently increased or decreased changes in transcript abundance in Hdac1-deficient embryos at 12 hpf, 18 hpf and 27 hpf. More than half of these genes are implicated in CNS development and down-regulated in Hdac1-deficient embryos. Within this set of co-regulated genes, we identified a core subset of 18 genes encoding sequence-specific DNA binding transcription factors, whose down-regulation could readily account for many of the neural specification defects that characterise the Hdac1-deficient CNS. Interestingly, the gene that exhibited the greatest-fold decrease in gene expression in *hdac1*-deficient embryos encodes a novel member of the methyltransferase-encoding NOL1/NOP2/Sun domain family of proteins, Nsun5, but nothing currently is known about the function of this protein.

Many of the 18 Hdac1-regulated CNS-specific transcription factor genes that were identified in our experiments play important roles in the specification of neuronal subtype identities in the spinal cord, brain and retina. Thus, *dbx2, lhx9, lbx1b, gsx1, bhlhe22, ascl1a, ascl1b *or their vertebrate orthologues have been implicated in neuronal specification within the spinal cord [22-26]. In the brain, *neurod, neurod4 *and *bhlhe22 *are are required for the production of cortical projection neurones [27,28], whilst upper motoneurones of the corticospinal tract depend on *fezf1 *[29] and specification of branchiomotor neurones of the mouse hindbrain requires the combined activities of *ascl1 *and *neurod4 *orthologues [30]. In the vertebrate retina, formation of amacrine cells is positively regulated by orthologues of *ascl1a/b, neurod, neurod4, foxn4, bhlhe22 *and *nr4a2 *[31-34]. Moreover, mammalian photoreceptor specification also requires *neurod*, *crx *and *rx *functions [35-37]. Taken together with these observations, our genome-wide expression analysis indicates that Hdac1 is deployed widely throughout the developing CNS to specify a wide variety of neuronal subtypes, by promoting the transcription of this core group of developmental regulatory genes. This conclusion was confirmed by our *in situ *hybridisation analysis of the expression patterns of proneural/bHLH genes *ascl1b, neurod4, *and *neurod, *along with *lhx9 *and the Notch ligand gene *dlb*. Transcription of these genes was considerably reduced throughout the Hdac1-deficient CNS, both at 12 hpf and at 27 hpf, and transient incubation of embryos in the HDAC inhibitor TSA between 10 hpf and 14 hpf almost completely extinguished their expression. By contrast, widespread expression of the neural transcription factor gene *sox2 *was not appreciably altered at 12 hpf, demonstrating that at this early stage, the reduced expression of *ascl1b, neurod4, neurod, dlb *and *lhx9 *in Hdac1-deficient embryos is not due to reduction in the pool of neural precursors, but rather the result of a failure to specify particular neuronal identities. Intriguingly, forced co-expression of the murine proneural gene *Ascl1 *(the orthologue of zebrafish *ascl1a *and *ascl1b*) with a small set of neuronal transcription factors in cultured murine fibroblasts, was recently shown to directly re-programme these cells to differentiate into distinct neuronal subtypes [38]. Remarkably, this group of collaborating murine transcription factors, defined by Vierbuchen et al., includes close relatives of *brn1 (Brn2), neurod (Neurod1), lhx9 (Lhx2) *and *nr4a2 (Nr2f1), *all of which we have found to exhibit stringent Hdac1-dependent co-expression throughout zebrafish neurogenesis. Interestingly, the murine Ascl1 and Brn1 proteins co-regulate a neurogenic programme by co-operative binding to a conserved DNA sequence motif in the *cis*-regulatory regions of *Delta *genes [39]. Thus, loss of *brn1 *and *ascl1b *expression in *hdac1 *mutant CNS could be responsible for the reduced expression of *dlb *that was also observed in *hdac1 *mutant zebrafish embryos. Overall, our analysis has uncovered a group of co-regulated neurogenic transcription factors that may function as a regulatory network driving neuronal patterning and differentiation in the CNS. It will now be of interest to investigate whether expressing particular combinations of these transcription factors in *hdac1 *mutant embryos can rescue defects of neuronal specification that are caused by loss of Hdac1 function.

Somewhat surprisingly, relatively few genes were identified in the time-resolved transcriptome analysis whose expression was robustly repressed by Hdac1 between 12 hpf and 27 hpf. Overall, 11 genes were identified, of mostly unknown functions, which exhibited a consistent, two-fold or greater increase in expression in Hdac1-deficient embryos across this period of development. Of these genes, 4 have been implicated previously in aspects of CNS development, raising the possibility that their increased transcription could contribute to the defects of neural development in Hdac1-deficient embryos. TRIM9, for example, encodes a brain-specific E3 ubiquitin ligase [40] and *fjx1 *encodes a Notch-regulated inhibitor of neuronal dendrite branching that is expressed in both the central and peripheral nervous systems [41]. Our previous work demonstrated that Hdac1 repressed the Notch target *her6 *at 26hpf and 33hpf [9], but *her6 *was not initially identified as an Hdac1-regulated gene in the time-resolved transcriptome analysis because its transcript abundance was not statistically significantly changed in 12 hpf and 18 hpf morphant embryos. Nevertheless, whole mount *in situ *hybridisation to Hdac1ATG1 morphant embryos revealed that *her6 *expression was appreciably increased in the hindbrain and optic vesicles at 12 hpf, which was confirmed in TSA-treated embryos, and that the increased *her6 *expression in the hindbrain persisted in 18 hpf *hdac1 *morphants. Thus, we conclude that Hdac1 attenuates *her6 *expression in restricted regions of the CNS from early stages of neurogenesis onwards.

### The role of Hdac1 in promoting transcription of genes required for CNS development

A recent microarray-based study of HDAC function in the differentiation of mouse retinal explants revealed that transcription of genes involved in promoting photoreceptor specification, such as *Crx*, *Neurod4 *and *Neurod, *as well as *Otx2 *and *Nrl *was rapidly suppressed within 3 hours of administering TSA to retinal explants [42]. Moreover, attenuation of *Crx *and *Nrl *expression by TSA was independent of a requirement for protein synthesis, implying a direct role for HDAC enzymes in promoting the transcription of these genes. We observed a similarly rapid and near complete extinction of *ascl1b, neurod4, dlb, neurod *and *lhx9 *expression, when 10 hpf embryos were incubated in TSA. Interestingly, many HDACs, including HDAC1, are abundant at the promoters of active genes in mammalian T lymphocytes, and closely associated with both HATs and phosphorylated RNA Polymerase II in the transcribed regions of these genes [7]. In yeast, HDACs biochemically oppose HAT functions by removing acetylation marks from chromatin at active genes, which facilitates their HAT-mediated re-acetylation in subsequent cycles of re-transcription. Accordingly, in yeast HDAC mutants, genes that are embedded within hyperacetylated chromatin are transcriptionally impaired [4-6], and moreover, similar observations have been made in mammalian T-cells [7]. Thus, it seems possible that some of the hyperacetylated histones we observed in *hdac1 *mutant zebrafish embryos are associated with genes whose transcription has been impaired by loss of Hdac1 function. The finding that Hdac1 is stably and specifically associated with sequences within the promoter region and first exon of *ascl1b *in 12 hpf and 27 hpf zebrafish embryos, transcription of which is Hdac1-dependent throughout this period, identifies *ascl1b *as a novel *in vivo *direct target of Hdac1. To our knowledge, this is the first such direct *in vivo *target to be defined for Hdac1 in vertebrate embryos. Taken together, our results suggest that Hdac1 could promote transcription of this proneural gene directly, by removing acetyl modifications from transcription unit-associated histones to enable their transcription-coupled re-acetylation, and/or by maintaining the *ascl1b *promoter in a deacetylated, H3K4 methylated, transcriptionally poised state. Indeed, the fact that global H3K4 methylation levels were unchanged by loss of *hdac1 *function (Figure 1) suggests that Hdac1 may bind to promoters within H3K4 methylated chromatin of zebrafish embryos, thus poising them for activation [7]. In view of the fact that our experiments were performed on whole embryos, it is possible that Hdac1 also binds to the *ascl1b *promoter in non-neuronal cells, although the significance of such possible interactions is unclear, as no ectopic expression of *ascl1b *was observed in Hdac1-deficient embryos. Future studies will aim to identify the DNA sequences within the *ascl1b *promoter, and the cognate DNA binding proteins, which recruit Hdac1 to this target gene, and determine whether these interactions are specific for the neuronal lineage in the developing CNS. We also intend to investigate how the distribution of epigenetic modifications across the genome is regulated by Hdac1.

Our *in vivo *chromatin immunoprecipitation studies detected no interactions between Hdac1 and the DNA sequences immediately upstream and downstream of the transcription start sites of *neurod4, neurod, deltab *and *her6*. Whilst it is possible that these genes are regulated by Hdac1 indirectly, it is also conceivable that Hdac1 binds directly to other *cis*-regulatory elements that are located further away from the transcription start sites of these genes. Another possibility is that the level of Hdac1 binding to these promoters *in vivo *may be below the threshold for detection in the chromatin immunoprecipitation assay. Despite the lack of Hdac1 binding to *her6 *promoter sequences, this gene remains a candidate direct target for direct repression by Hdac1, as Hdac1 repressed *her6 *expression in restricted regions of the CNS from 12 hpf onwards. Indeed, it remains possible that Hdac1-mediated repression of *her6 *facilitates the transcription of proneural genes such as *ascl1b *independently of the physical interaction between Hdac1 protein and the *ascl1b *promoter that we describe here. More extensive studies of Hdac1 binding to the chromatin in which these and other genes are embedded, and identification of proteins that recruit Hdac1 to its direct target genes, will allow the Hdac1-regulated genes identified by our transcriptome analysis to be evaluated further as candidate direct targets for Hdac1 binding. This information will help to define better the functional interrelationships between components of the Hdac1-regulated genetic network that promotes neurogenesis.

## Conclusions

We demonstrate that Hdac1 is an epigenetic regulator that governs the global levels of histone acetylation and H3K9 methylation during zebrafish development. Using a sensitive, in-depth, time-resolved transcriptome analysis of the *in vivo *function of Hdac1 during embryogenesis, we defined a principal requirement for Hdac1 to positively regulate the co-ordinated expression of 18 sequence-specific DNA binding transcription factor genes with known roles in neural specification and patterning, including several proneural bHLH genes. Chromatin immunoprecipitation analysis of candidate Hdac1 direct target genes in developing zebrafish embryos identified stable and specific binding of Hdac1 protein to the promoter of the proneural gene *ascl1b*. Although it is possible that this binding of Hdac1 to *ascl1b *occurs in non-neuronal cells, our results show that transcription of *ascl1b *is exquisitely sensitive to loss of *hdac1 *function, implying a role for Hdac1 in promoting *ascl1 *transcription in the neuronal lineage. Taken together, our results suggest that in addition to its well-documented functions in transcriptional repression, Hdac1 may also facilitate the direct transcriptional activation of target genes during vertebrate embryogenesis. In the developing CNS, this role could underpin the transcriptional poising of Hdac1 target genes encoding neuronal fate determinants, where these genes are silent but competent for transcriptional activation in response to neural specification and patterning signals.

## Methods

### Zebrafish stocks

*hdac1*^hi1618 ^mutant zebrafish were maintained at University of Sheffield. Individual embryos were genotyped using primers for *hdac1 *(Hdac1F: 5'-GGC AGG CGC AGG CTG TAA TT-3'; Hdac1 Intron1R: 5'-GGC TAA ACC CGG CTA ACA AT-3'); the 3' Long Terminal Repeat of the MLV vector (MLV 3LTR F: 5'- AAA GAC CCC ACC TGT AGG TTT G-3') [43]. Animal care and use was in accordance with the UK Animals (Scientific Procedures) Act 1986.

### Morpholino microinjection and drug treatment of embryos

Morpholino sequences were as follows: Hdac1ATG1: 5'-ttg ttc ctt gag aac tca gcg cca t-3'; Hdac1SPL1: 5'-ata ttc tta ccg tca taa taa tag c-3'; Standard Control (human β-globin): 5'-cct ctt acc tca gtt aca att tat a-3'; Hdac1 Mismatch control: 5'-ttg ctc gtt gag aac tct gca caa t-3'. 1-2nl of 0.3 mM morpholino solution in milli-Q water was microinjected into embryos at the 1-2-cell stage.

Trichostatin A (TSA) was dissolved in DMSO to 3 μM and added to E3 medium to a final concentration of 1 μM. 10 hpf wild-type embryos were incubated in E3 medium containing 1 mM TSA for four hours until 14 hpf, then samples were fixed and analysed by *in situ *hybridisation

### Whole-mount in situ hybridization and immunostaining of embryos

Whole-mount in situ hybridisation was performed using standard procedures.

For immunostaining with anti-Hdac1 antibody (abcam ab41407), embryos were fixed overnight, dehydrated in methanol and stored at -20°C. After rehydrating and permeabilizing with acetone at -20°C for 7 minutes, embryos were blocked in PBS containing 0.5% Triton-X, 1% DMSO, 1% BSA and 2% sheep serum (PBDTss) for 2 hours at 4°C and incubated with Hdac1 antibody (ab41407, 1:100, abcam) overnight. The next day, embryos were rinsed in PBDTss and incubated with alexa488-conjugated rabbit IgG (1:500, Invitrogen) before mounting for confocal microscopy.

### Western Blotting analysis of protein samples

200 μg of zebrafish embryo protein extract, corresponding to 5 whole 24hpf embryos, was separated by SDS-PAGE, transferred to Nitrocellulose (Amersham), and incubated with the following antibodies: anti-Hdac1 (ab41407, abcam), Pan-acetyl lysine (gift from C. Crane-Robinson, University of Portsmouth [44], H3acetylK9 (ab4441, abcam), H3dimethlyK9 (ab1220, abcam), H3trimethlyK4 (ab8580, abcam), H3 (ab1791, abcam). Signals were visualized using Horse Radish Peroxidase-conjugated secondary antibodies and the ECL system (GE Healthcare).

### RNA extraction, microarray hybridisation and gene expression analysis

40-60 embryos were treated with RNAlater (Ambion) and RNA was extracted using TRIzol. Traces of DNA were removed with RNase-free DNase I and RNA was purified on RNeasy columns (QIAGEN). Cy-dye (Perkin Elmer)-labelled amino Allyl-Modified aRNA probes (Ambion Amino Allyl MessageAmp II) were synthesised and hybridized to a custom Agilent 4 × 44 K array. Hybridizations were scanned using the standard Two-Color Microarray-Based Gene Expression Analysis Protocol (publication number G4140-90050; Agilent Technologies). The custom 44 K design contained a 22 K Agilent probe set together with a 16 K Sigma-Compugen probe set and bespoke oligo list [20]. In total the array contained probes corresponding to 18636 unique Unigene clusters and approximately 2000 additional Ensembl-annotated genes.

#### Experimental Designs

For the comparative analysis of 27 hpf wild-type and *hdac1 *mutant embryos, duplicate biological samples for each genotype were analysed in technical duplicate and with dye swaps at the probe labelling stage, giving total of 8 separate microarray datasets. For the analysis of morphant transcriptomes, biological duplicate batches of embryos injected with Hdac1ATG1, Hdac1SPL1, Hdac1Control and Standard Control morpholinos were all compared together in one microarray experiment, and for the time-resolved analysis of the Hdac1ATG1 transcriptome, biological triplicate batches of Hdac1ATG1 and Standard Control morphant embryos were compared at each time point.

To analyse the hybridisation data, the probe intensity values for each scanned Agilent chip was uploaded to the Rosetta Resolver system (Rosetta Biosoftware). Data from duplicates (technical, biological and dye-swap) were combined to give a combined fold-change and an associated p-value [45]. In all experiments, a p-value threshold of p < or = 10^-5 ^was used to define a probe as being significantly differentially expressed between two samples. The selection of this p-value as appropriate was determined by previous error-modelling and is an especially stringent criterion of significance [20]. Datasets were compared using Rosetta Resolver tools and selected data were then exported. Unannotated probes were annotated using homology searches (nBLAT, Ensembl) against the zebrafish genome and associated cDNAs or GenScan sequences were interrogated with BLAST searches. The microrray datasets used in our analyses have been deposited into GEO with Accession Number GSE26710.

Gene Ontology analysis was carried out using eGOn (v2.0, NTNU Gene Tools). Probe lists of significantly Hdac1-regulated genes (p < 10^-5^) were compared to a master probe list comprising all probes on the array in a Master-Target analysis to identify particular Gene Ontology classes that were over- or under-represented in the Hdac1-regulated list [46].

Hierarchical Array Clustering analysis was carried out using Cluster3.0 [21] and visualised in Java Tree view [47].

### Chromatin Immunoprecipitation and real-time quantitative PCR

For each batch of chromatin, three hundred wild-type (AB), *hdac1 *mutant, wild-type sibling, or morphant embryos were enzymatically dechorionated at 12 hpf or 27 hpf. Embryos were fixed immediately in 1.5 mM ethylene glycolbis[succinimidyl succinate] (EGS; Sigma-Aldrich) for 45 minutes, followed by 1.5% formaldehyde for 20 minutes [48]. Glycine (0.125 M) was added to quench the formaldehyde and embryos were washed in ice cold PBS. Embryos were deyolked [49] and homogenised on ice in extraction buffer with protease inhibitors. Chromatin was sheared by sonication to give DNA fragments of approximately 300-700 bp in size. Sonicated samples were centrifuged at 12,000 × g at 4°C for 30 minutes, and insoluble material was discarded. The supernatant was incubated at 4°C for 3 hours with 6 μg of either anti-Hdac1 antibody (Abcam ab41407) or control IgG antibody (Abcam ab46540). 80 μl of protein G magnetic beads (Pierce) were added to each sample and the samples rotated at 4°C overnight. Beads were washed six times with RIPA buffer at 4°C and bound complexes were eluted from the beads in Elution Buffer at 65°C for 20 minutes with vortexing. Cross-links were reversed overnight at 65°C and DNA fragments were purified by treatment with RNase A, followed by proteinase K digestion, phenol:chloroform:isoamyl alcohol extraction and ethanol precipitation. Quantitative real-time PCR (QPCR) of immuno-precipitated DNA was used to detect DNA sequences around the transcription start sites (+1) of *ascl1b*: between -785bp and +790 bp; *dlb*: between -964bp and +1018 bp; *neurod4*: between -803bp and +856 bp; *her6*: between -838bp and +825 bp. QPCR analyses were performed in triplicate and DNA abundance was normalised against Input values.

## Authors' contributions

VTC conceived the study; MRMH, ASG, HPS and VTC designed the experiments. MRMH and ASG performed the experiments; MRMH, ASG, HPS and VTC analysed the data. VTC, MRMH and ASG drafted the manuscript. All authors read, revised and approved the text of the final manuscript.

## Supplementary Material

Additional file 1 **Identification of Hdac1-regulated genes using morpholino knock down of *hdac1***. (A) Histograms of gene expression in Hdac1ATG1 and Hdac1SPL1 morphant embryos, measured against the Standard Control morphant common reference. Using a p-value of <10^-5 ^as cut-off for statistical significance, 7117/43427 microarray probes were identified that exhibited altered expression in Hdac1ATG1 morphants, of which 2557 probes exhibited >2-fold increased or decreased transcript abundance. By comparison, 16638/43427 probes were found to exhibit altered expression in Hdac1SPL1 morphants, of which 4391 probes exhibited >2-fold increased or decreased transcript abundance. Interestingly, however, whereas the Hdac1SPL1 morphant transcriptome exhibited many more differentially regulated genes overall than the Hdac1ATG1 transcriptome, 122 genes exhibited an 8-fold or greater change in expression as a result of the Hdac1ATG1 morpholino, as compared to 56 genes in the Hdac1SPL1 morphants. (B) Array cluster analysis of the transcriptomes of *hdac1 *mutant, Hdac1ATG1 and Hdac1SPL1 morphant embryos at 27 hpf. Cluster Tree depicts the degrees of similarity between datasets for all probes on each of individual arrays used and was carried using Cluster 3.0 analysis programme. Specific array IDs and the Hdac1 sample used are indicated. Remarkably, two of the *hdac1 *mutant technical duplicate datasets (10020_1 and 10020_3) cluster more closely with two of the Hdac1ATG1 biological duplicates (10085_1 and 10011_1) than they cluster with their dye-swap duplicates (10020_2 and 1002_4). The Hdac1SPL1 morphant (Splice) datasets cluster less closely with *hdac1 *mutant data, mostly clustering with Hdac1 Control morphant (HCo) data. Click here for file

Additional file 2 **Distribution of Gene-Ontology terms within the gene expression profile of Hdac1-deficient embryos**. (A) Biological process (GO:0008150), (B) Molecular function (GO:0003674) and (C) Cellular component (GO:0005575) categories. Only those Gene Ontology classes that exhibit an enrichment of Hdac1-regulated probes and have a p-value of 0.01 or less are shown. The distance of the Name of each Gene Ontology Term from the left-hand border of the table indicates the hierarchical position of the Term within the Gene Ontology framework. Hdac1-regulated genes are defined as those that exhibited increased or decreased expression in *hdac1*-deficient embryos, in the microarray experiments described in Figure 3.4. Gene Ontology classes that are directly related to the regulation of transcription are highlighted in purple. Click here for file

Additional file 3 **The majority of genes exhibiting robust Hdac1-dependent gene expression are involved in CNS development**. List of 84 probes that are consistently differentially expressed >2-fold in Hdac1ATG1 and Standard Control morphants at each of the three distinct time points, including their associated Unigene identifier and gene name. Fold-changes are listed for all three morphant time points and the *hdac1 *mutant fold change at 27 hpf is also indicated. Orange signifies probes corresponding to transcripts that are specifically expressed in the CNS or have a CNS-oriented function. Red labels indicate probes corresponding to transcripts that did not exhibit statistically significant differential expression greater than 1.2-fold in *hdac1 *mutant and wild-type sibling embryos, and are therefore likely to represent gene expression changes resulting from off-target effects of the Hdac1ATG1 morpholino. Click here for file

Additional file 4 **Efficient immunoprecipitation of Hdac1 protein from embryonic chromatin by anti-Hdac1 antibody**. Crosslinked, sonicated chromatin was prepared from 12 hpf zebrafish embryos, then incubated with anti-Hdac1 antibody and negative control IgG. Immune complexes were precipitated with Protein G-agarose and both immunoprecipitated proteins and unbound proteins were analysed by SDS-PAGE and Western blotting with the anti-Hdac1 antibody, with an equivalent sample of input chromatin run alongside. All Hdac1 protein in the input sample was recovered in the anti-Hdac1 immunoprecipitate, whereas none of the Hdac1 protein was immunoprecipitated by the negative control IgG and it remained in the unbound fraction. Click here for file
