# Supplementary figures and images for: The epigenetic regulator Histone Deacetylase 1 promotes transcription of a core neurogenic programme in zebrafish embryos
Source: BMC Genomics. 2011 Jan 12;12:24. doi: 10.1186/1471-2164-12-24 (PMC3032698; doi:10.1186/1471-2164-12-24)

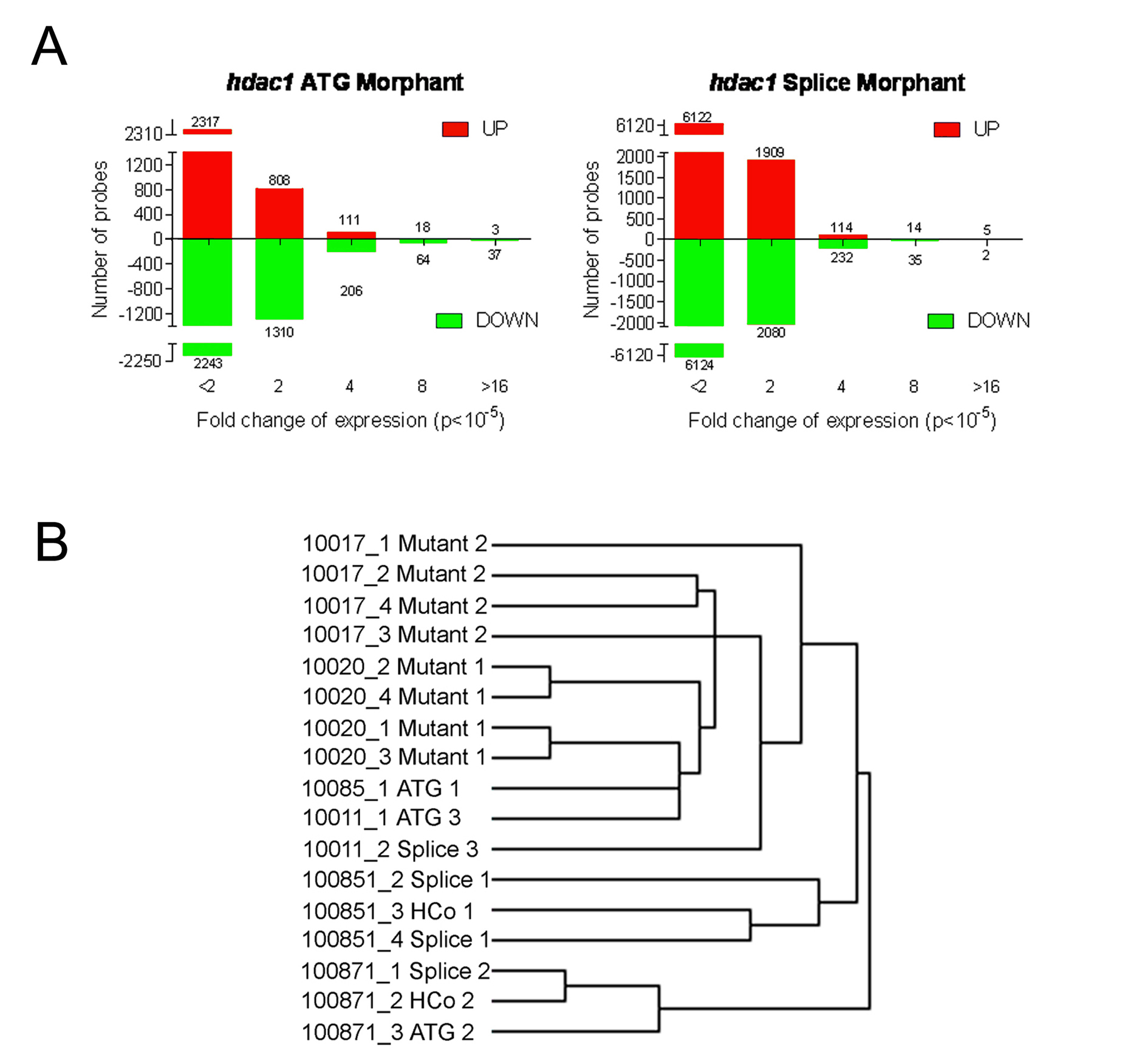

Supplement: Additional file 1 — Identification of Hdac1-regulated genes using morpholino knock down of hdac1. (A) Histograms of gene expression in Hdac1ATG1 and Hdac1SPL1 morphant embryos, measured against the Standard Control morphant common reference. Using a p-value of <10-5 as cut-off for statistical significance, 7117/43427 microarray probes were identified that exhibited altered expression in Hdac1ATG1 morphants, of which 2557 probes exhibited >2-fold increased or decreased transcript abundance. By comparison, 16638/43427 probes were found to exhibit altered expression in Hdac1SPL1 morphants, of which 4391 probes exhibited >2-fold increased or decreased transcript abundance. Interestingly, however, whereas the Hdac1SPL1 morphant transcriptome exhibited many more differentially regulated genes overall than the Hdac1ATG1 transcriptome, 122 genes exhibited an 8-fold or greater change in expression as a result of the Hdac1ATG1 morpholino, as compared to 56 genes in the Hdac1SPL1 morphants. (B) Array cluster analysis of the transcriptomes of hdac1 mutant, Hdac1ATG1 and Hdac1SPL1 morphant embryos at 27 hpf. Cluster Tree depicts the degrees of similarity between datasets for all probes on each of individual arrays used and was carried using Cluster 3.0 analysis programme. Specific array IDs and the Hdac1 sample used are indicated. Remarkably, two of the hdac1 mutant technical duplicate datasets (10020_1 and 10020_3) cluster more closely with two of the Hdac1ATG1 biological duplicates (10085_1 and 10011_1) than they cluster with their dye-swap duplicates (10020_2 and 1002_4). The Hdac1SPL1 morphant (Splice) datasets cluster less closely with hdac1 mutant data, mostly clustering with Hdac1 Control morphant (HCo) data. [file 1471-2164-12-24-S1.JPEG]

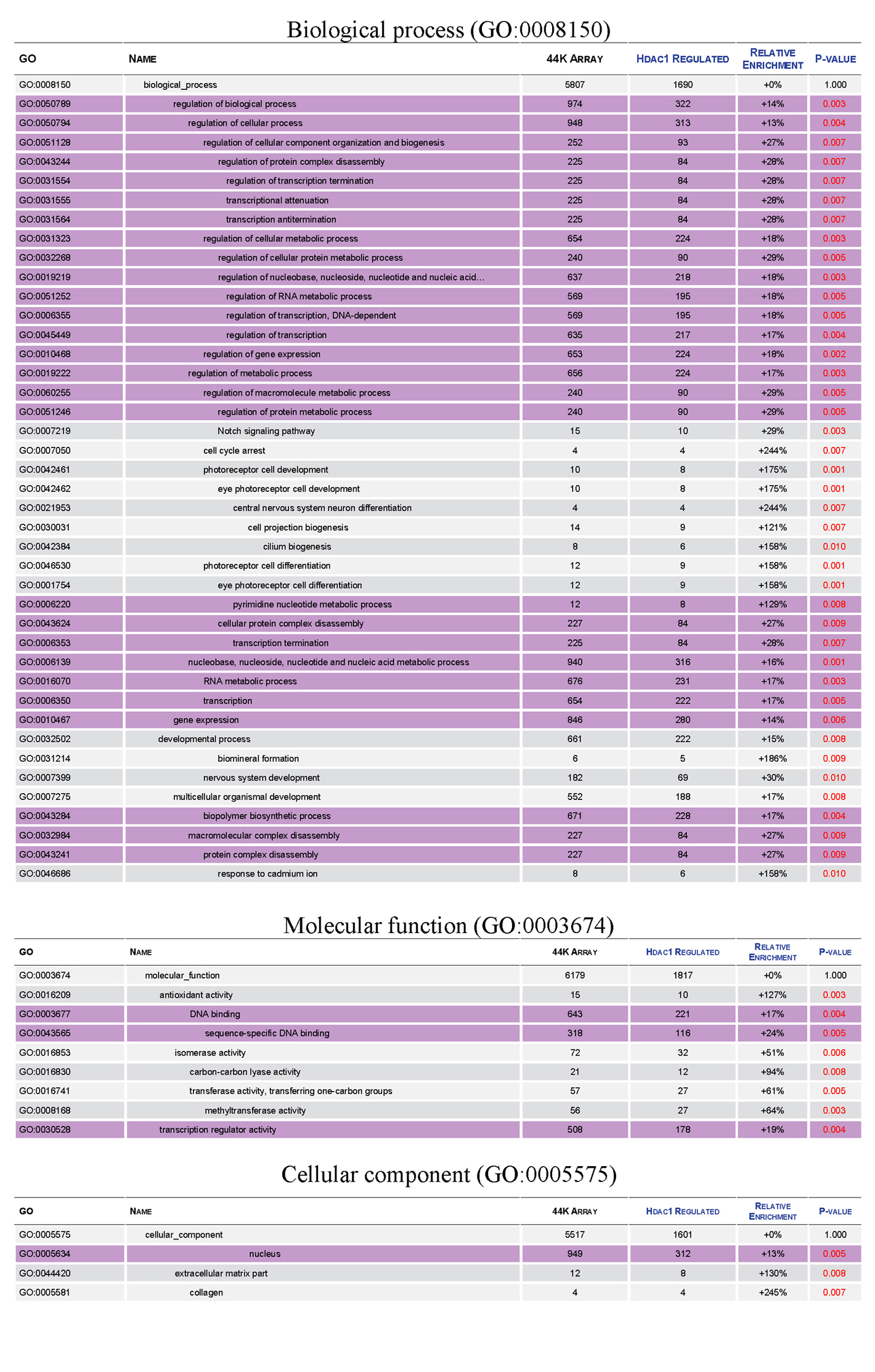

Supplement: Additional file 2 — Distribution of Gene-Ontology terms within the gene expression profile of Hdac1-deficient embryos. (A) Biological process (GO:0008150), (B) Molecular function (GO:0003674) and (C) Cellular component (GO:0005575) categories. Only those Gene Ontology classes that exhibit an enrichment of Hdac1-regulated probes and have a p-value of 0.01 or less are shown. The distance of the Name of each Gene Ontology Term from the left-hand border of the table indicates the hierarchical position of the Term within the Gene Ontology framework. Hdac1-regulated genes are defined as those that exhibited increased or decreased expression in hdac1-deficient embryos, in the microarray experiments described in Figure 3.4. Gene Ontology classes that are directly related to the regulation of transcription are highlighted in purple. [file 1471-2164-12-24-S2.JPEG]

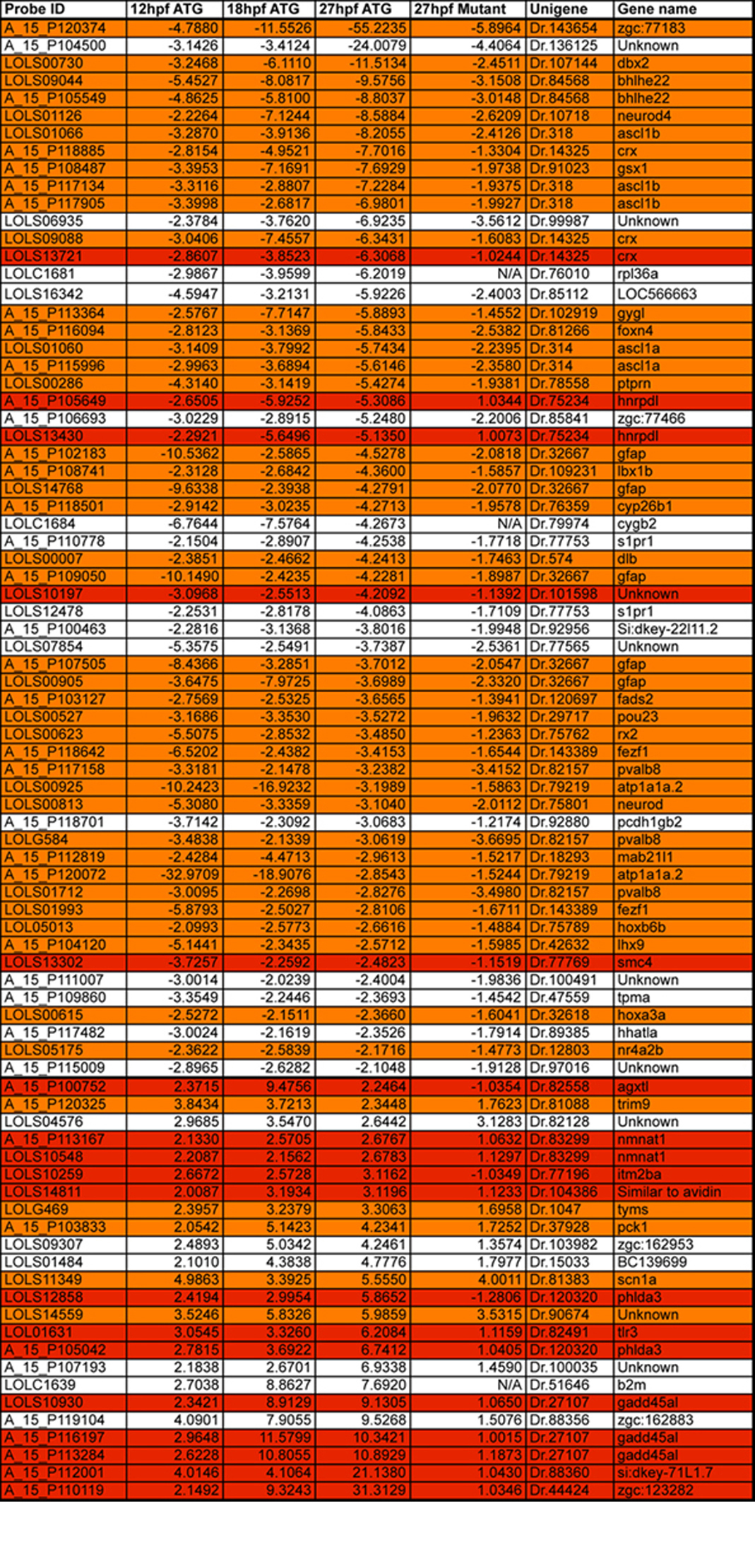

Supplement: Additional file 3 — The majority of genes exhibiting robust Hdac1-dependent gene expression are involved in CNS development. List of 84 probes that are consistently differentially expressed >2-fold in Hdac1ATG1 and Standard Control morphants at each of the three distinct time points, including their associated Unigene identifier and gene name. Fold-changes are listed for all three morphant time points and the hdac1 mutant fold change at 27 hpf is also indicated. Orange signifies probes corresponding to transcripts that are specifically expressed in the CNS or have a CNS-oriented function. Red labels indicate probes corresponding to transcripts that did not exhibit statistically significant differential expression greater than 1.2-fold in hdac1 mutant and wild-type sibling embryos, and are therefore likely to represent gene expression changes resulting from off-target effects of the Hdac1ATG1 morpholino. [file 1471-2164-12-24-S3.JPEG]

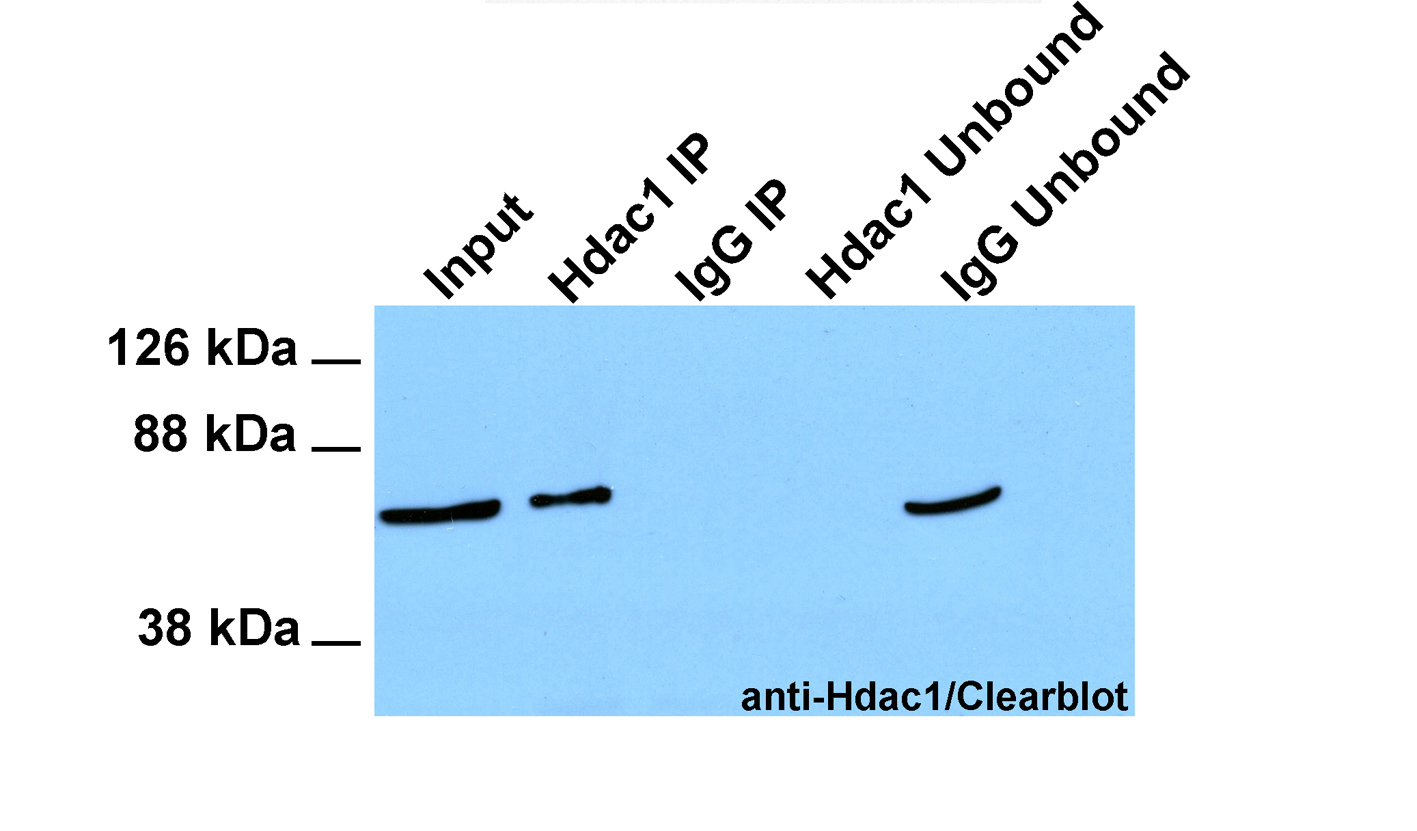

Supplement: Additional file 4 — Efficient immunoprecipitation of Hdac1 protein from embryonic chromatin by anti-Hdac1 antibody. Crosslinked, sonicated chromatin was prepared from 12 hpf zebrafish embryos, then incubated with anti-Hdac1 antibody and negative control IgG. Immune complexes were precipitated with Protein G-agarose and both immunoprecipitated proteins and unbound proteins were analysed by SDS-PAGE and Western blotting with the anti-Hdac1 antibody, with an equivalent sample of input chromatin run alongside. All Hdac1 protein in the input sample was recovered in the anti-Hdac1 immunoprecipitate, whereas none of the Hdac1 protein was immunoprecipitated by the negative control IgG and it remained in the unbound fraction. [file 1471-2164-12-24-S4.JPEG]
